# Experimental evolution, genetic analysis and genome re-sequencing reveal the mutation conferring artemisinin resistance in an isogenic lineage of malaria parasites

**DOI:** 10.1186/1471-2164-11-499

**Published:** 2010-09-16

**Authors:** Paul Hunt, Axel Martinelli, Katarzyna Modrzynska, Sofia Borges, Alison Creasey, Louise Rodrigues, Dario Beraldi, Laurence Loewe, Richard Fawcett, Sujai Kumar, Marian Thomson, Urmi Trivedi, Thomas D Otto, Arnab Pain, Mark Blaxter, Pedro Cravo

**Affiliations:** 1 Institute for Immunology and Infection Research, School of Biological Sciences, University of Edinburgh, Edinburgh, UK; 2 Centre for Immunity, Infection and Evolution, School of Biological Sciences, University of Edinburgh, Edinburgh, UK; 3 Centro de Malaria e Outras Doenças Tropicais/IHMT/UEI Biologia Molecular, Universidade Nova de Lisboa, Lisbon, Portugal; 4 Centro de Malaria e Outras Doenças Tropicais/IHMT/UEI Malaria, Universidade Nova de Lisboa, Lisbon, Portugal; 5 Institute for Evolutionary Biology, School of Biological Sciences, University of Edinburgh, Edinburgh, UK; 6 Centre for Systems Biology at Edinburgh, School of Biological Sciences, University of Edinburgh, Edinburgh, UK; 7 The GenePool, School of Biological Sciences, University of Edinburgh, Edinburgh, UK; 8 Pathogen Genomics Group, Wellcome Trust Sanger Institute, Hinxton, Cambridgeshire, UK; 9 Computational Bioscience Research Center, Chemical and Life Sciences and Engineering Division, King Abdullah University of Science and Technology, Thuwal 23955-6900, Kingdom of Saudi Arabia; 10 Division of Genetics and Genomics, The Roslin Institute and R(D)SVS, University of Edinburgh, Roslin, UK

## Abstract

**Background:**

Classical and quantitative linkage analyses of genetic crosses have traditionally been used to map genes of interest, such as those conferring chloroquine or quinine resistance in malaria parasites. Next-generation sequencing technologies now present the possibility of determining genome-wide genetic variation at single base-pair resolution. Here, we combine *in vivo *experimental evolution, a rapid genetic strategy and whole genome re-sequencing to identify the precise genetic basis of artemisinin resistance in a lineage of the rodent malaria parasite, *Plasmodium chabaudi*. Such genetic markers will further the investigation of resistance and its control in natural infections of the human malaria, *P. falciparum*.

**Results:**

A lineage of isogenic *in vivo *drug-selected mutant *P. chabaudi *parasites was investigated. By measuring the artemisinin responses of these clones, the appearance of an *in vivo *artemisinin resistance phenotype within the lineage was defined. The underlying genetic locus was mapped to a region of chromosome 2 by Linkage Group Selection in two different genetic crosses. Whole-genome deep coverage short-read re-sequencing (Illumina^® ^Solexa) defined the point mutations, insertions, deletions and copy-number variations arising in the lineage. Eight point mutations arise within the mutant lineage, only one of which appears on chromosome 2. This missense mutation arises contemporaneously with artemisinin resistance and maps to a gene encoding a de-ubiquitinating enzyme.

**Conclusions:**

This integrated approach facilitates the rapid identification of mutations conferring selectable phenotypes, without prior knowledge of biological and molecular mechanisms. For malaria, this model can identify candidate genes before resistant parasites are commonly observed in natural human malaria populations.

## Background

The molecular basis of drug resistance in malaria parasites and its evolution in time and space can be investigated, and possibly controlled, once the genes and specific mutations involved have been identified. However, despite intense investigations of human malaria parasites, few mutations have been unambiguously linked to drug resistance phenotypes. The most direct evidence for novel genetic markers comes from classical genetic studies that require no prior knowledge regarding mode of action or resistance [1]. For example, linkage analysis of a genetic cross in *Plasmodium falciparum *identified the locus containing *pfcrt *[2,3], the determinant of chloroquine resistance. This facilitated the characterisation of selective sweeps [4] driven by the world-wide use of chloroquine, and generated insights into the molecular basis of resistance [5,6]. Genetic studies have been extended to consider smaller gene effects, such as those underlying quinine susceptibility, by quantitative trait loci analysis [7]. However, there are experimental and ethical factors that constrain the use of *P. falciparum *for genetic studies. For example, it has sometimes proved difficult to generate drug-resistant *P. falciparum *mutant clones *in vitro *[8] and in these cases it has been necessary to await the appearance of resistant parasites in field samples. Also, although three genetic crosses between *P. falciparum *parasites have been performed and analysed [9-11] for genes involved in drug resistance or erythrocyte invasion, these require primate hosts and are therefore expensive and demand stringent ethical approval. Fortunately, the experimental tractability of the rodent malaria *P. chabaudi *presents a number of advantages, as follows.

**Figure 1:**
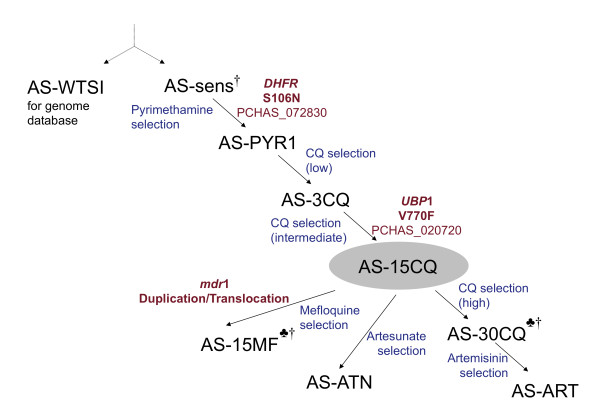
**AS lineage of drug resistant *P. chabaudi *parasite clones**. The cloned isolate AS-sens is sensitive to pyrimethamine (PYR), chloroquine (CQ), mefloquine (MF), artemisinin (ART) and artesunate. After passage in mice in the presence of PYR (single dose) and subsequently after multiple passage with increasing sub-lethal doses of CQ, MF, ART or artesunate (blue), surviving parasites were cloned to give AS-PYR1, AS-3CQ, AS-30CQ, AS-15MF, AS-ATN and AS-ART [13-16,49]. AS-15MF, AS-30CQ and AS-ATN were derived from an uncloned line, AS-15CQ [14]. ^♣ ^Two genetic backcrosses, between the genetically distinct drug-sensitive clone AJ and two members of the AS lineage (AS-15MF, AS-30CQ), were subjected to Linkage Group Selection (LGS) analysis. ^† ^Clones AS-sens, AS-30CQ and AS-15MF were subjected to whole-genome re-sequencing. Two specific gene mutations (red) that underlie PYR or ART resistance are shown at positions that indicate their origin within the lineage. Specific amino-acid substitutions refer to gene predictions in *P. chabaudi*. The predicted relationship between AS-sens and AS-WTSI is indicated as co-descendants of the ancestral AS isolate.

Experimental evolution of the rodent malaria *P. chabaudi *[12] previously produced an isogenic lineage comprising several genetically stable drug resistant clones, genetically related to a drug-sensitive clone (AS-sens, Figure 1), by the repeated *in vivo *passage of parasites in the presence of increasing but sub-curative doses of the drugs pyrimethamine, chloroquine, mefloquine and artemisinin derivatives [13-16]. In general, the sequence of drug resistance in this lineage reflects the historical pattern of selection of human malaria parasites by drug prophylaxis and treatment. Comparisons between wild-type and isogenic mutant parasites are especially informative: direct associations (between genotype and phenotype) can be observed in the absence of additional genetic diversity.

In order to map genes conferring selectable phenotypes, *P. chabaudi *has also been used to develop Linkage Group Selection (LGS) [17], where recombinant parasites from genetic crosses are analysed *en masse*; *i.e*. without the need to investigate the genotypes and phenotypes of individually cloned recombinant parasites. LGS scans the genome for 'selection valleys'; regions of selection where the proportion of alleles from a drug-sensitive parent are reduced in the uncloned drug-treated progeny of a genetic cross, relative to those in an untreated population. This approach has been used to map genes underlying pyrimethamine resistance [18] and strain-specific immunity [19,20] in *P. chabaudi*, and growth rate in *P. yoelii *[21].

Now, the advent of next-generation sequencing technology [22] makes possible whole-genome re-sequencing of mutant clones [23,24]. Because an annotated *P. chabaudi *AS-sens whole-genome assembly has recently been made available [25], a comprehensive whole genome re-sequencing of clones of the AS-lineage (Figure 1) will define all of the genetic variation arising within the lineage, identify mutations in selection valleys and, thereby, advance our understanding of the relationships between genetic mutation, drug response and selection.

This paper integrates these evolutionary, genetic and genomic approaches to specify the specific mutation underlying *in vivo *resistance to artemisinin. We demonstrate the evolution of artemisinin resistance in the *P. chabaudi *AS lineage, use LGS to map an underlying gene in two independent genetic crosses and re-sequence the complete genomes of the wild-type AS parasite and two artemisinin resistant mutants. We conclude that a single point mutation in the gene *ubp1*, encoding a de-ubiquitinating enzyme, confers artemisinin resistance in this *P. chabaudi *lineage.

## Results

### Experimental evolution of artemisinin resistance in *P. chabaudi*

**Figure 2:**
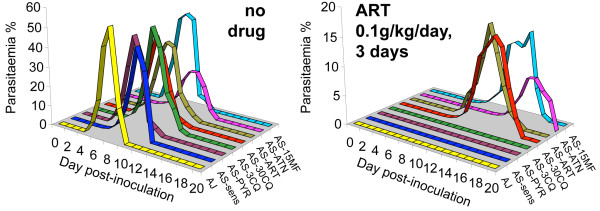
**Artemisinin resistance phenotype**. Artemisinin responses of four resistant and three susceptible AS clones (Figure 1) and AJ, mean of 3 mice. AS-30CQ, AS-ART, AS-ATN and AS-15MF showed artemisinin resistance. AS-3CQ, AS-PYR1, AS-sens and AJ are sensitive.

The strategy presented here exploits a comprehensive lineage of genetically related parasites (Figure 1), comprising drug-resistant mutants previously generated [13-16] by the sequential passage of parasites in mice treated with individual drugs (including artemisinin and artesunate) and the intermittent fixation of mutations by cloning. We determined the artemisinin responses of the genetically distinct cloned isolate AJ, and seven clones of the AS lineage when grown *in vivo *in mice (Figure 2). AS-sens, AS-PYR1, AS-3CQ and AJ all failed to establish detectable parasitaemias after ART treatment (100 mg kg^-1 ^for 3 days) whereas AS-30CQ, AS-15MF, and AS-ART gave detectable parasitaemias on day 8 or 9 post infection and peak parasitaemias of about 20% at some point between days 12 to 15. AS-ATN gave detectable parasitaemias on day 12, peaking at 10% during days 16 to 17. AJ, AS-sens, AS-PYR-1 and AS-3CQ were deemed artemisinin-sensitive because no parasites were detected at any time during post-treatment follow-up. Conversely, because artemisinin treatment failed for AS-30CQ, AS-15MF, AS-ART and AS-ATN, these parasites were classified as artemisinin-resistant. It was therefore predicted that mutations conferring artemisinin resistance were selected between AS-3CQ and AS-30CQ, AS-ATN or AS-15MF; interestingly, before parasites were ever exposed to artemisinin derivatives. AS-30CQ parasites had only been exposed to pyrimethamine and chloroquine, while AS-15MF had also been exposed to mefloquine. These data suggest that the gene mutations underlying artemisinin resistance may have pleiotropic effects. For example they may affect responses to other drugs such as chloroquine and mefloquine.

### Whole-genome genetic analysis of artemisinin resistance

**Figure 3:**
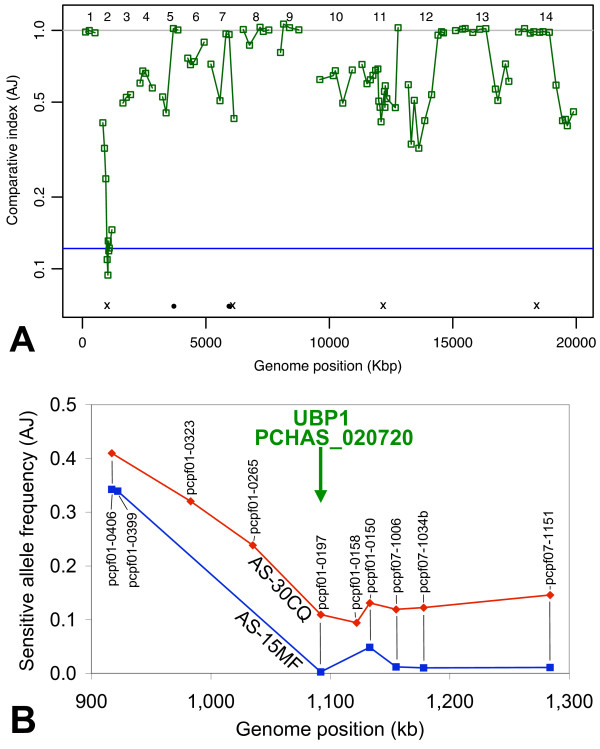
**Artemisinin resistance genetics**. **A**, Genome-wide scans of LGS of AS-30CQ × AJ backcross (under ART treatment) with quantitation of parental alleles by pyrosequencing [26]. Comparative Index (AJ) is the proportion of alleles in the population from sensitive parent AJ, after drug-selection, relative to the proportion measured in untreated hosts. Absence of selection would be represented by a Comparative Index of 1. Genome position indicates the mapped position (kb) of ~90 markers in the genome of *P. chabaudi *(Additional File 1). Data points (open green squares) are each mean of 3 independent determinations. Horizontal line (blue) indicates the threshold for rejecting null hypothesis in simulation analysis (p < 0.05, Additional File 2). Positions of mutations appearing in both AS-30CQ and AS-15MF as defined by Solexa genome re-sequencing are indicated at the bottom (x = point mutation; circle = indel). Note one sole point mutation on chr02 (*ubp1*) and one point mutation on chr07 (*dhfr*). Chromosome numbers are indicated (top). **B**, Position of ART-selection valley on chr02 as determined from two independent LGS scans of two genetic crosses (AS-30CQ × AJ, red; AS-15MF, × AJ blue) and the position of the mutated gene (*ubp1*, PCHAS_020720, green arrow). Pyrosequencing assays shown for whole chromosome (pcpfxx-yyyy, denotes *P. chabaudi*-specific assays at loci homologous to *P. falciparum *chrxx, location yyyy Kbp).

In order to map the genetic loci underlying artemisinin resistance, we analysed genetic crosses between either AS-30CQ (or AS-15MF) (both resistant parasites) and the genetically distinct drug-sensitive cloned isolate AJ, using LGS. The uncloned progeny of a genetic backcross (see Methods) between AS-30CQ and AJ (AS-30CQ × AJ) were treated with artemisinin (100 mg kg^-1^, 3 days). A genome-wide library of ~100 pyrosequencing assays [26] was then used to measure the proportions of AJ and AS alleles (single nucleotide polymorphisms, SNPs) at pre-mapped loci dispersed across the genome (Additional File 1) in both the drug-treated and the untreated populations. A single dominant selection valley was obtained on chr02 (Figure 3A). For instance, the AJ allele of the pcpf01-0197 marker here was reduced from 92.1% in the untreated population to 10.1% in the drug-treated population as shown in a detailed profile of selection on chr02 (Figure 3B). Except for loci on chr02, the proportions of AJ alleles of most markers in both the selected and unselected populations of parasites were generally high, possibly reflecting the predominance of AJ alleles in the backcross, the small proportion of parental AJ genotypes (drug-sensitive) and/or the existence of many AJ loci contributing small fitness effects in this experimental system. Factors such as these render the selection of AS alleles on chr02 even more striking.

Because apparent genetic associations can arise by chance, we used two statistical approaches to test the significance of this selection valley (Methods, Additional File 2). Firstly, the deepest selection valley from each of 500 simulations (Additional File 3) of the experimental design were used to estimate the probability (null hypothesis) that the depth of an observed selection valley was the result of random processes working in combination with selection at minor loci for resistance (p-value for no major gene = 0.026, Figure 3A, Additional Files 4, 5). Secondly, the non-parametric Mann-Whitney U-test was used to evaluate the probability that the allele frequency observed under drugs is significantly different from the random scatter of allele frequencies observed in the absence of drugs. The stringency of the statistical analysis was increased incrementally by reducing the AJ allele frequencies (AF-reduction) in untreated parasites (p < 0.001 at 50% AF-reduction, Additional File 6). Even so, the Mann-Whitney U-test underestimates the significance of the chr02 selection valley because the selection valley extended beyond the 5 consecutive loci used to compute significance (7 linked assays gave low AJ proportions, 4 of which give p < 0.05). Also, chr02 was the most dominant selection valley when a different genetic cross (AS-15MF × AJ) was selected with 100 mg kg^-1 ^ART (data not shown) and the profile of selection on chr02 was similar to that of the AS-30CQ × AJ cross (Figure 3B). In this case, the AJ frequency of marker pcpf01-0197 was reduced from 54.2% to 1.0% (Mann-Whitney, p < 0.001 at 25% AF-reduction).

### Whole genome re-sequencing of ART resistant parasites

The dominant chr02 selection valley indicates that both AS-15MF and AS-30CQ bear a mutation conferring artemisinin resistance in this locus. Previously, a mutation (V2728F, formerly V770F)) in a ubiquitin-specific protease (or de-ubiquitinating enzyme) gene (*ubp1*, PCHAS_020720) on chr02 was proposed to be linked to artemisinin resistance [27] but the presence of other mutations in this locus was not investigated. Here therefore, the Illumina^® ^Solexa platform [22] was used to define a comprehensive inventory of mutations arising within the *P. chabaudi *drug-resistant lineage (Methods, Additional File 7) and specifically, to determine the presence of other mutations on chr02. We sequenced the drug-sensitive clone AS-sens and the resistant clones AS-15MF and AS-30CQ (Table 1). 36 - 41 base single reads were aligned to the complete and annotated AS-sens reference genome sequence (AS-WTSI) [25] using MAQ [28] and SSAHA2 [29] mapping software packages, producing mean -fold coverage of ~ 39 (AS-sens), ~54 (AS-15MF) or ~77 (AS-30CQ). AS-WTSI comprises the majority of the genome assembled into 14 chromosomes, plus a small amount of sequence in contigs not assigned to any chromosome. The assigned chromosomal data contains all of the genes that have orthologues in *P. falciparum *(in large syntenic blocks [30]) plus some *P. chabaudi*- and rodent-specific genes and expanded gene families primarily located at sub-telomeric ends of assembled chromosomes. Candidate SNPs in AS-sens, AS-30CQ or AS-15MF relative to the AS-WTSI reference sequence were automatically called by MAQ and SSAHA2 algorithms. This process filters out unreliable, poor quality SNP calls on the basis of their associated quality indices and properties (*e.g*. read coverage, Phred-like scores, uniqueness and mapping quality, see Methods and Text S1). Lineage-specific candidate mutations were then identified by 'filtering out' SNPs specified for AS-30CQ (or AS-15MF) with those also called for AS-sens. Because artemisinin resistance arises after AS-3CQ, here we identify only those mutations that are present in *both *AS-30CQ and AS-15MF. This set of shared mutations will include those conferring resistance to artemisinin, as well as those conferring resistance to pyrimethamine and chloroquine.

**Table 1:** Solexa genome re-sequencing - summary statistics.

	**AS-30CQ**	**AS-15MF**	**AS-sens**
	
Passage number	71 AS-30CQ	38 AS-15MF	12407 AS-sens
Number of lanes	6	6	4
Read type	single-end	single-end	single-end
Read length (bases)	41	36	36
Nominal fold coverage	30X	30X	20X
	**% reads mapped to reference sequence**		
	
MAQ	97%	90%	91%
SSAHA2	97%	90%	93%
	**- fold coverage**		
	
MAQ	77.91	54.21	39.28
SSAHA2	75.86	53.97	39.48

MAQ and SSAHA2 analysis revealed only four shared point mutations, located on chr02, chr07, chr11 and chr14 (Table 2, Additional File 8). Their positions relative to the chr02 selection valley are shown in the genome-wide genetic scan of artemisinin selected progeny (Figure 3A). Three of the four point mutations (chr02, chr07, chr11) are missense mutations in genes with known orthologues in other *Plasmodium *spp. The remaining point mutation is located within an intergenic region on chr14. All four point mutations were confirmed by di-deoxy sequencing and their origin within the AS lineage was determined (Figure 1). Two point mutations (on chr02 and chr07) corresponded to genetic changes previously described in the AS lineage. The point mutation on chr07 in the gene encoding dihydrofolate reductase (*dhfr*, PCHAS_072830) corresponds to a S106N substitution. It arises between AS-sens and AS-PYR1 and underlies resistance to PYR [31,32]. The mutation on chr02 is the missense mutation (V2728F) in *ubp1*. It arises between AS-3CQ and AS-30CQ [27] (Figure 1).

**Table 2:** Point mutations common to *both *AS-30CQ and AS-15MF.

Chr	**Ref. seq**. (AS-WTSI)	AS-sens	AS-30CQ and AS-15MF	Gene ID	Substitution	Gene abbreviation	Annotation
2	C	C	A	PCHAS_020720	V2728F	***ubp1***	de-ubiquitinating enzyme
7	G	G	A	PCHAS_072830	S106N	***dhfr***	dihydrofolate reductase
11	G	G	T	-	non-synonymous	-	-
14	T	T	G	-	intergenic	-	-

This analysis of high-quality SNPs also predicted three point mutations specific to AS-30CQ and one point mutation specific to AS-15MF, all on chromosomes other than chr02 (data not shown). The complete set of lineage-specific mutations is therefore extremely limited suggesting that a small number of mutations account for the evolution of multiple drug resistance phenotypes in this lineage. Their contribution to other drug-resistance phenotypes (such as chloroquine resistance) will be evaluated in future investigations.

Five further putative point mutations (in AS-30CQ) were identified by SSAHA2 but not by MAQ (data not shown). These putative mutations have markedly lower quality scores and low relative coverage. One mapped to a gene without a *P. falciparum *orthologue and was negated by di-deoxy sequencing. The other four mapped to sub-telomeric or unassigned contigs which have reduced sequence complexity, increased mapping ambiguity and inferior reference sequence quality. We were therefore unable to amplify products from these regions for di-deoxy sequencing.

Previous sequence analysis of the orthologues of four candidate genes (*atp6, crt*, *mdr1*, *tctp*) showed that these were not mutated in AS-ART [16]. As expected, the genome re-sequencing data presented here showed that there were no mutations in these genes in AS-30CQ or AS-15MF either.

A major effect gene conferring artemisinin resistance was mapped to chr02 by LGS analysis, as described in the previous section. If the V2728F *ubp1 *point mutation is the sole mutation identified on chr02 and if that mutation arose between the isogenic sensitive and resistant parasite, it must be considered the critical mutation conferring the resistance phenotype. It was therefore important to evaluate the possibility that a chr02 mutation had been missed in our analysis.

False negative calls (missed mutations) could be present in areas with insufficient read coverage and/or yielding poor mapping quality (*e.g*. regions containing low complexity or repetitive sequence). In order to evaluate the probable extent of these regions, we analysed *in silico *the proportion of the AS-WTSI genome sequence to which 36 - or 41 base reads could be mapped uniquely (Additional File 7). These values were 98.63%, and 98.80% respectively (Table 3). For the experimentally obtained data, 97.78 or 97.95% of the genome was sequenced at ≥ 5 read coverage for AS-sens and AS-30CQ respectively (Table 3). These metrics show that only a small percentage of the genome could not be re-sequenced with uniquely mapping reads. We also estimated the proportion of each chromosome sequence for which the experimental read coverage is low (≤10). These values range from 0.6% (chr09) to 3.8% (chr01) (Table 4). These data confirm that the genome re-sequencing was almost comprehensive and that false negatives will be confined to < ~ 3% of the genome. We therefore believe that the number of point mutations missed by our analysis is likely to be zero or a very small number, or otherwise restricted to *P.chabaudi*-specific expanded gene families, usually located at sub-telomeric loci. The read-coverage of all nucleotides up to 200 kbp either side of *ubp1 *was also investigated. Here, the proportion of bases with a read-coverage ≤ 10 reads in AS-30CQ was 0.63% (Table 4). Because a read-coverage of 10 exceeded that sufficient to detect a false positive SNP, and because the frequency of mutations across the genome in AS-30CQ is so low (~7 point mutations/~20 Mbases), these data confirm that a false negative within the selection valley is highly unlikely.

**Table 3:** Unique mapping of reads to AS-WTSI reference sequence.

	number of bases mapped		% of genome sequence (WTSI) mapped			
	*in silico *(unique)		experimental (unique)			
			AS		AS-30CQ	
			> = 5	> = 10	> = 5	> = 10
**36 mer**	18,574,867	98.63	97.78	95.88		
**41 mer**	18,606,976	98.80			97.95	96.96

*In silico *analysis shows the number of bases and percentage of genome which can be uniquely mapped by sequence reads (equivalent to theoretical maximum). Experimental analysis shows the percentage of genome actually mapped by such reads, at different read-coverages (> = 5, > = 10) in AS-sens or AS-30CQ. AS-sens and AS-30CQ were analysed with 36 - and 41 base reads, respectively.

**Table 4:** AS-30CQ genome coverage by chromosome

Chromosome	% coverage >10
Chr01	96.16%
Chr02	98.92%
Chr03	96.32%
Chr04	97.48%
Chr05	97.71%
Chr06	98.04%
Chr07	96.61%
Chr08	99.27%
Chr09	99.39%
Chr10	98.11%
Chr11	98.78%
Chr12	98.88%
Chr13	98.74%
Chr14	98.84%
**Total (chrs only)**	**98.31%**
	
unassigned contigs	71.69%
	
*ubp1 *+/-200 kb on chr02	99.37%

*% of nucleotides sequenced by >10 reads

### Identification of insertions, deletions and copy number variants by Solexa re-sequencing

We applied MAQ and SSAHA2 to detect candidate insertions/deletions (indels) and copy number variants (CNVs). Full details of this analysis are given in Additional Files 7, 9. To summarise, we used SSAHA2 to predict small (≤ 3 bp) insertions and deletions (indels), but no such mutations were confirmed. Analysis of decreased or increased read coverage can be used to predict larger deletions and CNVs, respectively. A 34 bp deletion in an intergenic region of chr07 was confirmed by di-deoxy sequencing. A potential large (~1 kb) deletion at the right-hand end of chr05 within a rodent malaria-specific gene (PCHAS_051920) was also identified but unconfirmed because appropriate DNA fragments could not be amplified and sequenced, probably because of low-complexity genomic sequence. Specifically, there were no predictions of indels or CNVs on chr02, confirming that these mutation classes are unlikely to confer artemisinin resistance in AS-30CQ.

## Discussion

### Ubp1 mutation (V2728F) confers ART resistance

Various lines of evidence confirm that the V2728F *ubp1 *mutation confers artemisinin resistance. The *ubp1 *mutation mapped to the bottom of the selection valley in both genetic crosses analysed (Figure 3B). Solexa re-sequencing shows that this mutation is the only genetic variation between resistant and sensitive clones on chr02. Sanger dideoxy-sequencing confirmed that the wild-type V2697, V2728 *ubp*1 haplotype is carried by all four of the artemisinin-sensitive parasites (AS-sens, AS-PYR, AS-3CQ and AJ) while V2728F (in AS-30CQ, AS-ART and AS-15MF) or V2697F (in AS-ATN, formerly V739F) mutations [27] are borne by parasites with artemisinin resistance (Additional File 10 in this lineage of isogenic parasites. Of the eight point mutations identified within the AS lineage, those in u*bp1 *were the only ones acquired along with the artemisinin resistance phenotype (*i.e*. between AS-3CQ and AS-30CQ, AS-15MF, or AS-ATN).

The V2728F *ubp1 *mutation in the AS lineage, was previously identified [27] but its association with artemisinin resistance was not demonstrated because (i) the *ubp1 *mutation in AS-ART arose during the generation of AS-30CQ under chloroquine selection, and could not underpin the increase in artemisinin resistance in AS-ART relative to AS-30CQ, (ii) the selection valley on chr02 (in the AS-ART × AJ cross) was not sufficiently dominant with a 25 mg kg^-1 ^artemisinin selection, and (iii) the possibility that additional mutations in genes linked to *ubp1 *on chr02 could not be excluded. The present data resolve these ambiguities. Firstly, all clones (including AS-30CQ, AS-15MF, AS-ATN and AS-ART) with *ubp1 *mutations have artemisinin-resistant phenotypes (100 mg kg^-1^, 3 d). Secondly, the chr02 locus is strongly selected in two different additional independent crosses by 100 mg kg^-1 ^artemisinin. Three independent genetic crosses (using one of the artemisinin-resistant clones AS-30CQ, AS-15MF (both in this report) or AS-ART [27]) have now mapped the gene underlying artemisinin resistance to chr02. Thirdly, whole-genome re-sequencing confirmed that the point mutation in *ubp1 *is the only mutation on chr02.

The cloned parasite AS-ATN (Figures 1, 2) also has an artemisinin resistance phenotype but contains a different *ubp1 *mutation (V2697F) [27]. We propose that the V2697F *ubp1 *mutation underlies artemisinin resistance in AS-ATN too. Formal confirmation of this would require an LGS analysis to identify a chr02 selection valley in an AS-ATN × AJ cross (with respect to artemisinin, or artesunate), and genome re-sequencing of AS-ATN to confirm that there was no other mutation on chr02. We believe that both *ubp1 *mutations arose (in different parasites) during the production of the uncloned line AS-15CQ [14] from AS-3CQ under chloroquine selection (Additional File 11). This line was subsequently subjected to further selection by chloroquine [14], mefloquine [15] or artesunate [16]. Chloroquine and mefloquine are thought to have selected (to fixation, or near fixation) V2728F *ubp1 *parasites, while artesunate is thought to have selected V2697F *ubp1 *parasites. A full discussion of these issues is included elsewhere (Additional File 11)

The structural consequences of the V2728F *ubp1 *mutation in AS-30CQ (and V2697F *ubp1 *in AS-ATN) were predicted to be a reduction in de-ubiquitinating activity [27]. This would increase the proportion of ubiquitinated substrate, which may be destined for degradation through the 26 S proteasome. We suggest that reduced parasite susceptibility to artemisinin may involve increased turnover or altered trafficking of a specific protein or class of proteins (such as membrane transporters like *MDR1*, regulators of cell cycle etc.), or non-specific effects such as a general increased turnover of proteins damaged by oxidative stress

### The model - its generality, application and future development

The experimental integration of mutant selection, genetic linkage analysis using LGS, and genome re-sequencing is a powerful framework for the elucidation of genetic determinants without prior knowledge of the underlying biology of the phenotype. The model exploits the co-incidence between genetically defined selection valleys and isolated mutations identified by deep genome re-sequencing. Importantly, it may also confirm the lack of dominant contributions from additional genes elsewhere in the genome. The approach complements reverse genetic validation (transfection) of gene candidates which may be restricted to limited numbers of pre-defined genes and gene mutations (allelic replacements). Unfortunately, a well-developed transfection system is currently not available in *P. chabaudi*.

The approach is applicable to many recombining organisms for which genome sequence data and experimentally tractable selectable phenotypes are available. In *P. falciparum*, there remain difficulties in reliably generating genetically stable drug-resistant mutants and the practicality of performing genetic crosses [12]. Nevertheless, the approach described here may find applications in the study of human malaria. For example, a whole-genome tiling array was used to identify a large amplification event in an *in vitro *selected fosmidomycin-resistant *P. falciparum *mutant clone [33]. The amplified fragment included the gene encoding 1-deoxy-D-xylulose-5-phosphate reductoisomerase, the putative target of fosmidomycin. Deep re-sequencing technologies can add further resolution to such investigations. Importantly, in addition, genetic analyses such as LGS also map the functional relationship between gene locus and phenotype; critical when elucidating resistance to drugs for which the mode of action is not well understood.

Pyrosequencing assays were used here to estimate the proportions of AS and AJ parental alleles and to detect loci under selection. However, we envisage the future exploitation of deep short-read sequencing to rapidly define and quantitate parental SNPs in pooled recombinant parasites (from genetic crosses) before and after selection. We expect this to improve the detection and resolution of selection valleys in LGS experiments. It will also avoid the time required to develop pre-mapped quantitative parental markers, and permit the use of a greater range of species and strains.

### The genetic basis of multi-drug resistance

The comprehensive genome re-sequencing described here identified a small number of (mainly non-synonymous) mutations arising in the drug-resistance lineage of *P. chabaudi*. These data suggest that complex multi-drug resistant phenotypes evolved with the accumulation of a relatively small number of mutations, contrary to some previous speculations [14]. The question arises: are all of these mutations selected (to fixation or near fixation) by drugs, or are some mutations neutral and randomly fixed during cloning by stochastic processes? Future studies will address this question and evaluate the extent to which *ubp1 *and other mutations affect the responses of these parasites to other drugs such as chloroquine.

### Public health implications

The prediction of genes involved in drug resistance phenotypes has important public health implications, especially when candidate markers are proposed before resistance is commonly observed in the field, as is the case for the current study. For example, molecular genetic markers of resistance are required to monitor strategies used to contain the spread of resistant parasites. At present, we are vulnerable to the evolution of artemisinin resistance. Indeed, although artemisinin combination therapies continue to be widely effective, there have been a number of recent reports documenting reduced susceptibilities of malaria parasites to artemisinin derivatives *in vivo *and *in vitro*, especially in parts of South-east Asia, such as Western Cambodia [34-36]. Although genes such as the multi-drug resistance locus, *mdr1 *[37,38], or *pfatp6 *[39], encoding an ATP-ase (SERCA-type), have been proposed to contribute to artemisinin resistance, either these markers have yet to be confirmed or it is generally believed that they mediate small effects or reflect responses to the non-artemisinin partner drug [40,41]. At present therefore, there are no generally accepted validated markers for artemisinin resistance-mediated treatment failure.

The *ubp1 *mutations are the first genetic determinants to be shown to underlie a distinct change in *in vivo *artemisinin responses. The relevance of mutations or polymorphisms in the *ubp1 *orthologue (PFA0220w on *P. falciparum *chr01) to *in vivo *and *in vitro *artemisinin responses of *P. falciparum *parasites can now be addressed by characterising natural infections and by transfection studies in the laboratory. For example, a recent study of parasites from Western Cambodia [42] showed that there were no corresponding mutations in *ubp1 *in artemisinin-resistant *P. falciparum *parasites.

The data presented here show that, in *P. chabaudi*, a single point mutation confers a distinct shift in artemisinin responses. However, whether further mutations in *ubp1 *or other genes may confer additional shifts in artemisinin response is still uncertain. These possibilities can be addressed by the generation of multiple independently selected resistant clones of *P. chabaudi*, or by selecting mutants with higher levels of resistance.

Interestingly, artemisinin-resistance and V2728F *ubp1 *mutations arose in the *P. chabaudi *isogenic lineage before the parasites were exposed to artemisinin itself. Thus, *ubp1 *mutations may prove to play a role in multi-drug resistance, as is the case for *mdr1 *[43]. These relationships are the subject of ongoing investigations. Although a distinct change in artemisinin response is described here, observations that *in vitro *IC_50_s of artemisinin derivatives in *P. falciparum *are often correlated with those of structurally unrelated aminoquinolines [44-46] may indicate that similar processes occur in human malaria parasites.

The identification of *ubp1 *mutations will augment our understanding of artemisinin-resistance mechanisms by opening up new experimental approaches, such as the identification of proteins interacting with *ubp1*. This may clarify whether artemisinin resistance is mediated by the modulation of pre-existing drug resistance mechanisms or by interference with the pathway of artemisinin action.

Combination therapies have been proposed to reduce the probability of selecting (multi-) resistant parasites [47,48]. This rationale depends upon choosing component drugs with different mechanisms of resistance, and upon the absence of pre-existing resistance to one (or both) components. The future choice of optimal combinations may be guided by knowledge delivered using a predictive model such as that presented here.

## Conclusion

Experimental evolution of drug resistance, a genome-wide genetic linkage analysis (LGS) and whole genome deep re-sequencing have been used to define the mutation conferring artemisinin resistance in the malaria parasite, *P. chabaudi*. This system can be used for the rapid identification of candidate genetic markers of *in vivo *drug resistance, before resistance arises in the field. These data may be used to monitor, understand and prevent the evolution of drug resistance in the field and to choose component drugs in combination treatments designed to maximise drug longevity.

## Methods

### Parasite lines, maintenance, parasite preparation and DNA extraction

A number of *P. chabaudi *clones of the AS-lineage (Figure 1) were used [12-16]. They were routinely inoculated, passaged in CBA mice (4-6 weeks) and cryopreserved as previously described [49]. Parasites were prepared and DNA extracted as previously described [50], ensuring that host white cells were removed by CF11 cellulose (Whatman, UK) and Plasmodipur filters (Eurodiagnostica, Netherlands).

### Phenotyping, drug tests

Artemisinin drug tests for *P. chabaudi *parasites have been previously described [16,27]. Here we used 3 day treatments on day 1-3 post-inoculation (p.i.) (1 × 10^6 ^parasites) at the defined doses in CBA mice. Parasitaemias were estimated from a drop of blood from the tail in Giemsa stained thin blood smears as follows. Infected and uninfected RBCs were counted in up to a minimum of 2 representative microscope fields (approx 100 RBC/field). Counting of additional fields was stopped when > 20 infected RBCs had been counted (maximum 5 fields).

### Genetic crosses

The generation of recombinant progeny arising from genetic crosses between drug-sensitive (AJ) and drug-resistant (AS lineage) parasites has been previously described [13,15,18,27]. In the present experiments, back crosses of both AS-30CQ × AJ and AS-15MF × AJ against the sensitive AJ background were performed in order to maximise the numbers of independent recombinant parasites and to optimise the resolution of selection valleys. Previously generated cryopreserved cross progeny [14,15] were selected with different drug concentrations (including untreated) and the surviving parasites at day 5 to day 11 p.i. mixed in approximately equal proportion with sensitive (AJ) parasites prior to the genetic backcross procedure. 1 × 10^5 ^mixed parasites were inoculated into C57 mice for production of gametocytes. Parasites were transmitted on day 5 p.i. or day 6 p.i. through *Anopheles stephensi *mosquitoes, maintained as described previously [49]. Sporozoites arising in the mosquitoes were recovered by intraperitoneal injection, as previously described [13,15,18,27]. Asexual red blood cell forms were collected, pooled and used to inoculate CBA mice used for drug selection (LGS) experiments.

### Quantitative analysis of genetic crosses

We used pyrosequencing (Biotage) to determine the proportions of parental alleles at many polymorphic sites across the genome as previously described [26] and according to the manufacturer's methodology. In this study, which pre-dated the release of the full genome assembly and annotation, the position of individual markers within the *P. chabaudi *genome was defined as follows. The required position of markers within the *P. chabaudi *genome was mapped to orthologous genes in the *P. falciparum *genome using the rodent malaria synteny maps [30]. Suitable *P. falciparum *protein sequences were used to define *P. chabaudi *sequences in orthologous genes by TBLASTN analysis, with validation of adjacent genes in both species. SNPs between AS and AJ parasites were identified by amplification of nucleotide sequences from AS and AJ parasites, and assays developed using high-quality sequence from both clones. Details of the genome-wide set of assays are given in Additional File 1.

### Statistical analysis of genome-wide scans

**Overview **We developed and used two independent approaches to assess the statistical significance of putative selection valleys. The first is based on simulations of the experimental design and tests the null-hypothesis that resistance is caused by many loci of small effects and can therefore not be attributed to one or a few dominant selection valleys. The second approach compares observed and corrected allele frequencies in experiments with and without drug treatment using the non-parametric Mann-Whitney U-test. Both approaches have complementary strengths and weaknesses. Our main goal is to identify a statistically significant selection valley. The principles, methodologies, results, model and validations are detailed in Additional Files 2, 3, 4, 5, 6, 12.

### Genome re-sequencing analysis by the Illumina^® ^(Solexa) platform

Clones AS-sens, AS-15MF and AS-30CQ were re-sequenced by the Illumina^® ^(Solexa) platform with 36 - (AS-sens, AS-15MF) or 50 base (AS-30CQ) single reads (nominal coverage x20, x30, x30 fold respectively) (Table 1). For AS-30CQ, the final 9 base calls were universally of poor quality and were removed before mapping the remaining 41 base sequence strings. Individual sequence strings (reads) from each clone were aligned against an isogenic AS reference sequence (AS-WTSI, September 2009 assembly, obtained from the publicly available ftp site of the *P. chabaudi *AS genome sequencing project website at the Wellcome Trust Sanger Institute; ftp://ftp.sanger.ac.uk/pub/pathogens/P_chabaudi/) using two different software packages; MAQ (Mapping and Assembly with Quality) [28] and SSAHA2 (Sequence Search and Alignment by Hashing Algorithm) [29].

For SSAHA2 analysis, Solexa reads were mapped against AS-WTSI using the "pileup" command for single end reads with the appropriate options as described in ftp://ftp.sanger.ac.uk/pub/zn1/ssaha_pileup/ssaha_pileup-readme. Three output files (*.snp, *.ins and *.del) calling SNPs, insertions or deletions were combined into a single Artemis [51] - compatible file (*.gff) using a custom script that further selected SNPs based on a minimum read coverage that was set at "3" for all the clones, in this study. A second script was developed to compare the *.gff files of either of the two mutant clones against the output obtained for AS-sens. In this way, only mutations appearing within the AS-lineage were retained. Furthermore, a third custom script was used to filter out all the remaining SNPs that had ambiguous (or heterozygous) base calls, where the 'minority' base was represented in ≥20% of the total number of reads. The filtered files were then imported into Artemis which allowed the inspection, identification and evaluation of these mutations on the fully annotated genome. For example, mutations could be immediately identifed as intergenic or intragenic, synonymous or non-synonymous etc.

MAQ was used to call point mutations and compared to those made by SSAHA2. MAQ analysis was performed using the "easyrun" command in the MAQ software suite with default parameters, as described in http://maq.sourceforge.net. This generates a list of candidate SNPs for each clone as a 'cns.final.snp' file. These SNPs were subsequently filtered using custom scripts, as follows (see also Additional File 7). A minimum coverage of 3 reads was applied to call SNPs in all clones. Proposed SNPs with ambiguous calls were removed. The SNPs obtained for mutant clones were then filtered to remove those SNPs defined for AS-sens. The remaining SNPs are candidates for mutations within the AS lineage.

The identification of insertion/deletion mutations (indels) and CNVs was performed with SSAHA2 using two approaches, namely SSAHA2's internal algorithm for the identification of small indels (≤ 3bp) and a "comparative coverage" analysis, both described in detail in the Additional File 7. Visualisation of individual reads and coverage which is available in MAQ was used to confirm indels and CNVs identified by SSAHA2, giving increased confidence.

## Competing interests

The authors declare that they have no competing interests.

## Authors' contributions

KM, SB, AC, LR, RF, MT performed phenotype, genetic or genomic experiments and analysed data. AM, SK, UT, TO analysed Solexa data. DB, LL developed and performed statistical analysis. MB, AP supported genomic analysis. PH, PC conceived, designed, co-ordinated study, analysed and interpreted data. PH wrote the manuscript with assistance from other authors. All authors have read and approved the final manuscript.

## Supplementary Material

Additional file 1 **Inventory of pyrosequencing assays**. The approximate genome position (column M) of pyrosequencing assays (columns B-G) are based upon mapping to a previous assembly and annotation of AS-WTSI, using estimated lengths for telomeres, gaps between contigs etc. The locus designation pcxxpfyy-zzzz indicates that the marker maps approximately to *P. chabaudi *chromosome xx and homologous to a locus in P. falciparum zzzz kb along chromosome yy Click here for file

Additional file 2 **Statistics: methods, analysis and evaluation**. This file describes the statistical methods developed for evaluating whether selection at given loci is significant. Two methods are described and evaluated. Click here for file

Additional file 3 **Simulations - examples**. Four independent examples (#1, 9, 10, 12) of simulated genome scans of comparative index (marker (AJ, drug-sensitive parent) proportion in drug-treated population/ marker proportion in untreated population) v. genome positions of markers on 14 chromosomes (numbers at top) assuming null hypothesis of 10 resistance (AS advantage, red dots) and 10 fitness (AJ advantage, blue dots) loci are shown. The z-score (Additional File 2) of the deepest valley is calculated (bottom left). Additional File 4 plots the frequency of these z-scores after 500 such simulations. Note that, in contrast to experimental scans (Figure 3A), many simulations show multiple valleys, including minor valleys containing only neutral markers. Click here for file

Additional file 4 **Simulations - null distribution histogram**. The distribution of the lowest *z*-scores was observed in 500 simulations of selection of the AS-30CQ × AJ cross, using experimental marker positions. The null hypothesis assumed 10 loci for artemisinin resistance and 10 minor fitness alleles. The red point indicates the experimentally observed *z*-score (-2.364) for *chr2 *under artemisinin selection (Figure 3A) corresponding to a p-value of 0.0263. See Additional File 2 for details. Click here for file

Additional file 5 **Simulations - the inferred significance of observed deepest selection valleys**. These data are derived from the simulation approach. See Additional File 2 for full details. ^1 ^z-score observed in the experiments; ^2 ^p-value corresponding to the minimum z-score obtained experimentally; ^3 ^simulations for ART reproduce the experiment that led to the genome-wide scan in Figure 3A. Click here for file

Additional file 6 **Mann-Whitney analysis of genome-wide LGS**. Scan showing allele frequencies (proportion of AJ allele) from pyrosequencing after untreated infection (control, black triangles) and treated infection (artemisinin 100 mg/kg/day, 3 days, red diamonds) (n.b Figure 3A in main text uses Comparative Index (y-axis, defined in legend Figure 3) rather than allele proportion used here). Treatment is expected to bias AJ allele frequencies downward by removing the sensitive parent, AJ and recombinants free from resistance mutations. To correct for this and other factors (Text S1), control allele frequencies were reduced before testing potential selection valleys. Significant valleys are highlighted by blue circles (P < 0.001 in Mann-Whitney U-tests comparing a sliding window of 3-5 markers on the same chromosome (treated) with all untreated markers reduced by the percentage (R) indicated (AF-reduction); grey triangles show untreated frequencies resulting from a -25% shift). Positions of this strain's new mutations found by genome comparisons are indicated at the bottom (x = SNP; circle = indel). Chromosome numbers are given at the top. Genome position indicates physical mapping (Kbp) of 92 markers in the genome of *P. chabaudi *(Additional File 1). Data points are each the mean of 3 independent determinations. Click here for file

Additional file 7 **Solexa whole-genome re-sequencing - extended methods, results, analysis, evaluation and quality control**. This file includes an extended description of the analysis of the Solexa Illumina data. Click here for file

Additional file 8 **Point Mutations (shared by AS-30CQ and AS-15MF wrt AS-sens)**. This table specifies details of point mutations predicted to be common to AS-30CQ and AS-15MF. Click here for file

Additional file 9 **Indels and CNVs (shared by AS-30CQ and AS-15MF wrt AS-sens)**. This table specifies details of indels and CNVs predicted to be common to AS-30CQ and AS-15MF. Click here for file

Additional file 10 **Artemisinin, pyrimethamine responses and *ubp1*, *dhfr *mutations in clones of the AS lineage of *P. chabaudi***. This table summarises the pyrimethamine and artemisinin responses of various clones of the lineage and their genotype, with respect to *dhfr *and *ubp1*. Click here for file

Additional file 11 **AS-15CQ genotype and the origin of alternative *ubp1 *V2697F mutation in AS-ATN**. This file discusses a number of issues regarding AS-15CQ, its non-clonality and the origins of two mutations in *ubp1*. Click here for file

Additional file 12 **Validation of simulation - comparing simulation data and experimental data**. This file summarises data which validates the simulation approach to statistical analysis. Click here for file
