# Supplementary material for: Experimental evolution, genetic analysis and genome re-sequencing reveal the mutation conferring artemisinin resistance in an isogenic lineage of malaria parasites
Source: BMC Genomics. 2010 Sep 16;11:499. doi: 10.1186/1471-2164-11-499 (PMC2996995; doi:10.1186/1471-2164-11-499)
Supplement: Additional file 2 — Statistics: methods, analysis and evaluation. This file describes the statistical methods developed for evaluating whether selection at given loci is significant. Two methods are described and evaluated. [file 1471-2164-11-499-S2.DOC]

**Additional File 2**

**Statistical methods, analysis and evaluation**

**Approach 1: Simulating the experimental design**

**Principle** Statistical thresholds are established to determine whether a marker is under selection by analysing simulation models that mimic actual experimental designs. In principle we simulate LGS genome scans where drug resistance is *not* due to genes of large effect (null hypothesis, see below). In each of these simulations we record the deepest selection valley found in the simulation in order to construct a probability distribution of the depth of selection valleys for the simulated conditions. We then compare the depth of the experimentally observed selection valley with that distribution to determine the probability that the experimental observation could have been produced by the random noise and the small effects of many loci under the simulated model. The frequency with which a selection valley as deep as an experimentally observed one is found in such simulations can be taken as the probability that the experimentally observed valley is a false positive (i.e. it is caused by random variation of allele frequencies, or genes conferring only limited drug resistance).

**Methodological details** The genome scans for artemisinin (ART) resistance are simulated according to the known biology and adopted experimental procedures [12-16]. For the initial genetic crosses (AS-15MF or AS-30CQ with AJ), 600 haploid individuals are generated by randomly mating equal proportions of AS (drug-resistant) and AJ (drug-sensitive) parental strains. Simulating the backcross step (recombining the drug-selected population of the initial cross, with AJ) involves generating 12000 and 600 haploid individuals for the strains AS-30CQ and AS-15MF, respectively (values generated from estimated oocyst numbers in dissected mosquitoes). The simulated recombinant haplotypes are generated using genetic maps which are co-linear with physical maps (presented in the main text) assuming 15kb/cM [Martinelli *et al*., 2005, see end of file].

Asexual growth after recombination and before the measurement of allele frequencies allows for selection to occur. Each generated haplotype increases its number of individuals in proportion to the multiplicative fitness effects that are distributed across its genome. The increases in individuals for each clone are Poisson distributed. Selection affects the average proportion of individuals that succeed in replicating. This is modelled by a coefficient between 0 and 1 as established for each haplotype on the basis of its genotype and the drug treatment applied. Simulated replication (cloning) was stopped during the generation that most closely approaches 10^9^ individuals, as in *in vivo* protocols. Similarly, bottlenecks (transfers of parasites from mouse to mouse) are simulated by random sampling 10^6^ parasites (inoculum size) from the source population.

All allele frequencies in real data and in the simulations are converted to comparative indices (AJ allele frequency in treated population/AJ allele frequency in untreated population). These are used to compute the *z*-score, which measures the deviation of a data-point compared to that of the population:

*z = (x – μ) / σ,*

where *x* is the comparative index at the tested marker, and *μ* and *σ* are the mean and standard deviation of the comparative indices of all the markers in the genome scan.

Based on these results, the lowest *z*-scores are recorded in each simulation to compute a null distribution. The lower quantile to which an experimentally observed *z*-score is mapped on the null distribution represents the probability that it is a false positive. From each null distribution, the *z*-scores corresponding to the probabilities at 0.005, 0.01, 0.05 and 0.1 were extracted by the quantile function of R using its default parameters.

Simulations and analyses are implemented in R (http://www.R-project.org). The functions to simulate LGS experiments are available from author DB as an R package along with the script specific to the models constructed here.

**Null hypothesis** Each experimental design is simulated 500 times under the null hypothesis that drug resistance is caused by several genes of small effect that act multiplicatively (analogous to one used in QTL mapping by variance components [Williams and Blangero, 1999, see end of file]. Two scenarios are simulated. In the first one, 20 loci for drug resistance are distributed on the genome at random positions. The *combined* multiplicative effect of these genes is such that, under drug treatment, a pure AJ haplotype has 1% of the reproductive success of a pure AS strain (i.e. for 100 AS individuals that reproduce in each generation, one AJ individual is reproduced on average). In the second scenario only ten drug resistance genes are assumed to exist.

Since AJ is known to outgrow the drug resistant AS strain in the absence of drug treatment, both scenarios of selection include ten randomly distributed fitness loci, which reproduce the slower growth rate of AS compared to AJ in both the presence and absence of drugs. The combined multiplicative effect of these “fitness” genes causes the reproductive success of AS to be 20% of that of AJ in the absence of drug selection. The combination of drug sensitivity alleles from AJ and fitness disadvantage alleles from AS determines the relative growth rate of a haplotype, all relevant effective selection coefficients being computed multiplicatively.

**Results** Four examples of a simulation and the z-score of the deepest valleys are shown in Additional File 3.

A distribution of the deepest selection valleys from 500 simulations (10 minor loci, 10 fitness loci) is shown in Additional File 4. Additional File 5 reports the corresponding results for our observed LGS scan. The inferred p-values were estimated as follows

1. compute the comparative indices for loci of interest from Figure 3A (main text)
2. determine the corresponding *z*-scores, and
3. find the corresponding p-value from Additional File 5 to indicate the significance of the selection valley. The actual values were calculated using the quantile function in R.
4. We also determined the z-score corresponding to a p-value of 0.05. The CI value equivalent to this z-score is marked as a horizontal line on Figure 3A (main text).

**Model validation** Despite our attempts to be realistic, our simulation models necessarily simplify the underlying biology. Thus we wish to evaluate our models and the parameters that we have assumed, all of which could influence an LGS profile. To this end we compared the means and standard deviations of the allele frequencies from simulated LGS data to those obtained from the experimental scans. In our simulations and in real experiments, the means are affected by the relative fitness of one strain compared to the other while the standard deviations depend primarily on the effective number of dominant recombinant clones (data not shown).

As shown in Additional File 12, there is an acceptable agreement between experimental data and model simulations. Models where drug selection is weak or absent or where a weak growth disadvantage is assigned to the AS strain are rejected because they produce scans with means and standard deviations that are substantially different from the experimental observations. Although an exhaustive search for optimal parameter values in our simulations is beyond the scope of our study, Additional File 12 suggests that useful *z*-score thresholds may be computed for a reasonable range of parameter choices.

**Reporting of analysis in manuscript** For ART-selection of the AS-30CQ x AJ and AS-15MF x AJ backcross, the experimental comparative indices are displayed in Figure 3A. These plots also show the mapped positions of the mutations identified. A horizontal line represents the comparative index which gives rejection of the null hypothesis at p < 0.05. Any markers with a lower comparative index are thus adjudged to have a major influence on drug-resistance.

**Approach 2: Comparing untreated and treated allele frequencies directly**

**Principle** Here we attempt to compare the AJ allele frequencies (sensitive parent) in the untreated and drug-treated populations to identify sets of linked markers where the AJ allele frequency is significantly reduced after drug treatment. We used a non-parametric test which analyses the changes in the rank order of the selected allele frequencies in a genome-wide sample of unselected allele frequencies. In order to increase the stringency of this analysis, we consolidated up to 5 linked markers into a ‘bin’. We also introduced a strategy which further increases the stringency by reducing the values of AJ allele frequencies in the unselected control population by a constant amount *R* (‘AF-reduction’). This procedure makes selection valley recognition more robust. To see why, we need to consider the various causes for differences in allele frequencies between untreated and drug treated samples.

**Causes for differences in allele frequencies in LGS scans** We assume that a difference between treated and untreated allele frequencies can be caused by (i) experimental noise, including stochastic sampling of alleles during rescue, propagation and inoculation, (ii) deterministic selection on resistance loci that causes genuine selection valleys by reducing the frequency of sensitive AJ alleles, (iii) deterministic selection on fitness loci that causes genuine selection peaks by increasing the frequency of AJ alleles with a growth advantage in the absence of drugs (such a phase follows after each selection phase in LGS experiments) and (iv) the following complex sampling and selection process that can lead to a reduction of the mean and an increase of the variance of the AJ allele frequencies. The sensitive AJ clone is thought to bear a number of ‘fitness loci’ just as the resistant AS clone bears one or more resistant loci. If, after recombination, neutral markers are trapped by chance in clones with various combinations of one or more of these differently selected loci, then selection can drastically affect the allele frequency at the neutral locus. This can give the appearance of a spurious selection valley and result in an increase of the variance of allele frequencies after drug treatment when compared to untreated samples. This effect is particularly strong if few fitness loci exist and only few recombinants are produced during a backcross (data not shown). Likewise, clones that happen to carry no resistance alleles after recombination (AJ only) have no chance of surviving drug selection and will thus contribute to a reduction of the mean of observed AJ allele frequencies after selection when compared to allele frequencies without selection. For example, if only one resistance locus exists and there is no assortative mating, Mendel’s laws predict that 25% of all progeny are expected to have the genotype of the sensitive parent and would thus get removed after selecting for resistance.

To properly correct for these consequences of recombination with subsequent selection would require an almost complete knowledge of the corresponding genome that is studied by LGS. However approximate corrections can be attempted. One such approximate correction is the simulation method presented above. ‘Allele Frequency (AF) - reduction’, as outlined here, constitutes another such approximation.

Observations have shown that the sensitive (AJ) allele frequencies of treated samples are systematically lower than those of untreated samples. We assume that this is due to a reduction in the mean or a possibly additional increase in variance, as explained above. Here then, we apply a simple correction factor *R* to reduce the untreated AJ allele frequencies (‘AF-reduction’). This corrects a potential downward bias in the mean of allele frequencies in the selected samples and can also counter a potential increase in variance as long as this is not too extreme. The latter is possible because reducing the mean allele frequency in the untreated control population will increasingly remove spurious selection valleys which are frequently less deep than those that are genuine. This procedure consolidates many complicated processes into a single value that is impossible to compute *ab initio*, but has some desirable properties.

- All unknown details are consolidated in only *one* parameter without loss of information.
- The unknown parameter *R* (the size of a change in allele frequency) has an intuitive meaning and is limited to values between 0 and 100%, reducing the search space.

While this approach cannot deliver absolute certainties, it has proven useful in evaluating the significance of different selection valleys in both the same and different LGS experiments. The AF-reduction analysis generates a movable baseline to progressively reject minor selection valleys.

**Detailed methodology** We applied this analysis to the LGS plots shown in Additional File 6. We used the Mann-Whitney U-test to compare the untreated AJ allele frequencies (almost 100 data points) with a sliding window of 3-5 treated AJ allele frequencies to compute the probability that the mean of the sliding window is not different from the mean of the large population of untreated allele frequencies.

We chose the non-parametric unpaired Mann-Whitney U-test, which ranks the data to compute the corresponding probabilities, as it is free from assumptions about the nature of the underlying distribution. The non-parametric strategy allows us to gain generality and robustness at the expense of not using quantitative data which represents the *depth* of a selection valley. However, some of these losses are offset by using different values of *R* (see above). This allows us to identify selection valleys that remain statistically significant, even when the background AJ allele frequency is greatly reduced. Thus, strong selection valleys will continue to present significant p-values for increasing *R* values, while the significance of weaker valleys will disappear.

For the data summarised in Additional File 6 we

1. computed the null distribution by reducing all allele frequencies in the untreated populations by the defined value of *R*.
2. defined a ‘bin’ of linked allele frequencies for a focal marker, including a maximum of two markers before and two after it (provided that they are on the same chromosome, resulting in a minimum total of 3 markers per bin).
3. Conduct the test (in R use: wilcox.test with paired = FALSE and alternative = "greater"). Here we chose a p-value < 0.001 at a shift of *R* = 25.0%, 37.5% and 50.0% as a critical threshold.
4. For all markers in the genome, perform steps (ii) and (iii).

**Results** We applied this test to all datapoints in our LGS scans at *R* = 25%, 37.5% and 50%. We show the significant results as blue shapes in Additional File 6.

**References**

Martinelli A, Hunt P, Fawcett R, Cravo P, Walliker D, Carter R: **An AFLP-based genetic linkage map of *Plasmodium chabaudi chabaudi*.** *Malar J* 2005, 4:1-11

Williams J, Blangero J: **Power of variance component linkage analysis to detect quantitative trait loci.** *Ann Hum Genet* 1999, **63:**545-563
