# Supplementary figures and images for: Experimental evolution, genetic analysis and genome re-sequencing reveal the mutation conferring artemisinin resistance in an isogenic lineage of malaria parasites
Source: BMC Genomics. 2010 Sep 16;11:499. doi: 10.1186/1471-2164-11-499 (PMC2996995; doi:10.1186/1471-2164-11-499)

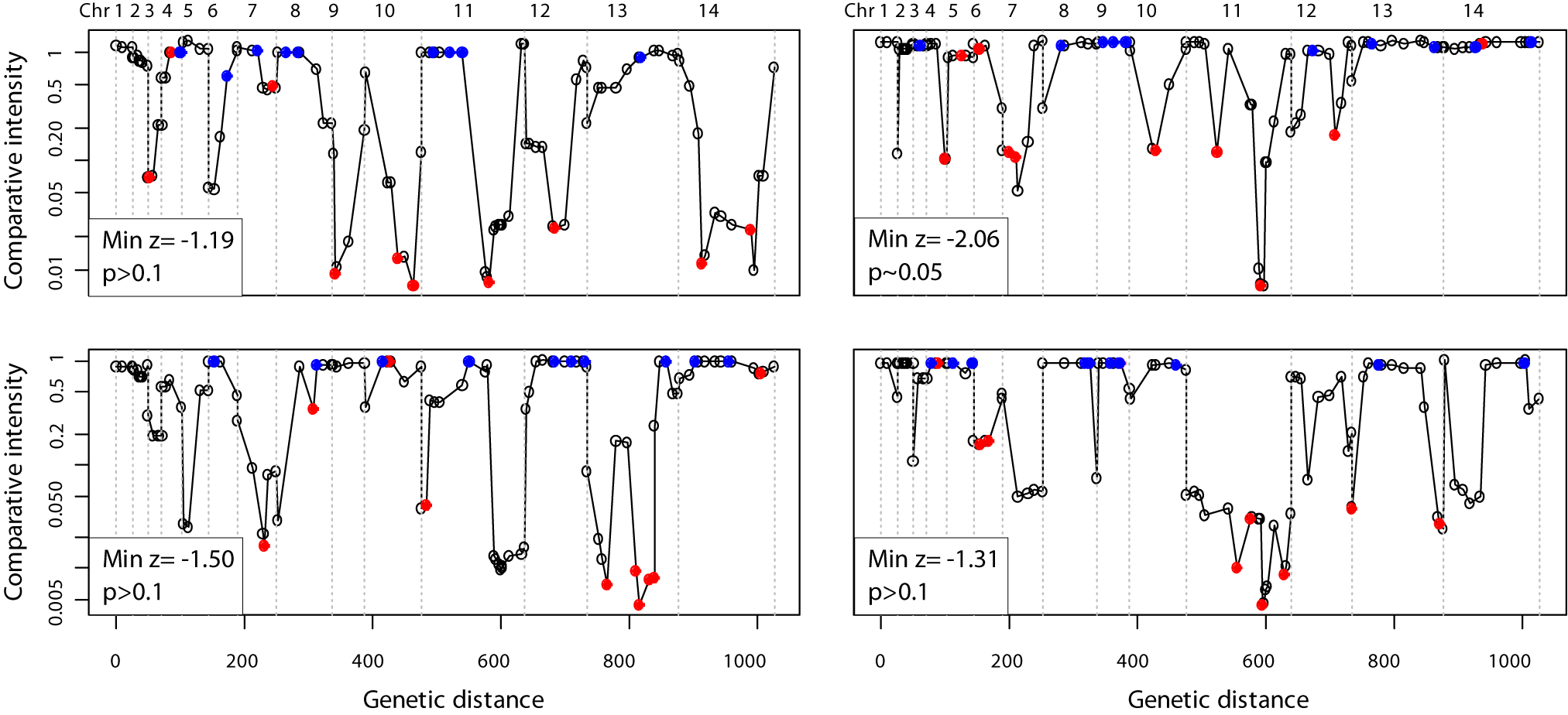

Supplement: Additional file 3 — Simulations - examples. Four independent examples (#1, 9, 10, 12) of simulated genome scans of comparative index (marker (AJ, drug-sensitive parent) proportion in drug-treated population/ marker proportion in untreated population) v. genome positions of markers on 14 chromosomes (numbers at top) assuming null hypothesis of 10 resistance (AS advantage, red dots) and 10 fitness (AJ advantage, blue dots) loci are shown. The z-score (Additional File 2) of the deepest valley is calculated (bottom left). Additional File 4 plots the frequency of these z-scores after 500 such simulations. Note that, in contrast to experimental scans (Figure 3A), many simulations show multiple valleys, including minor valleys containing only neutral markers. [file 1471-2164-11-499-S3.TIFF]

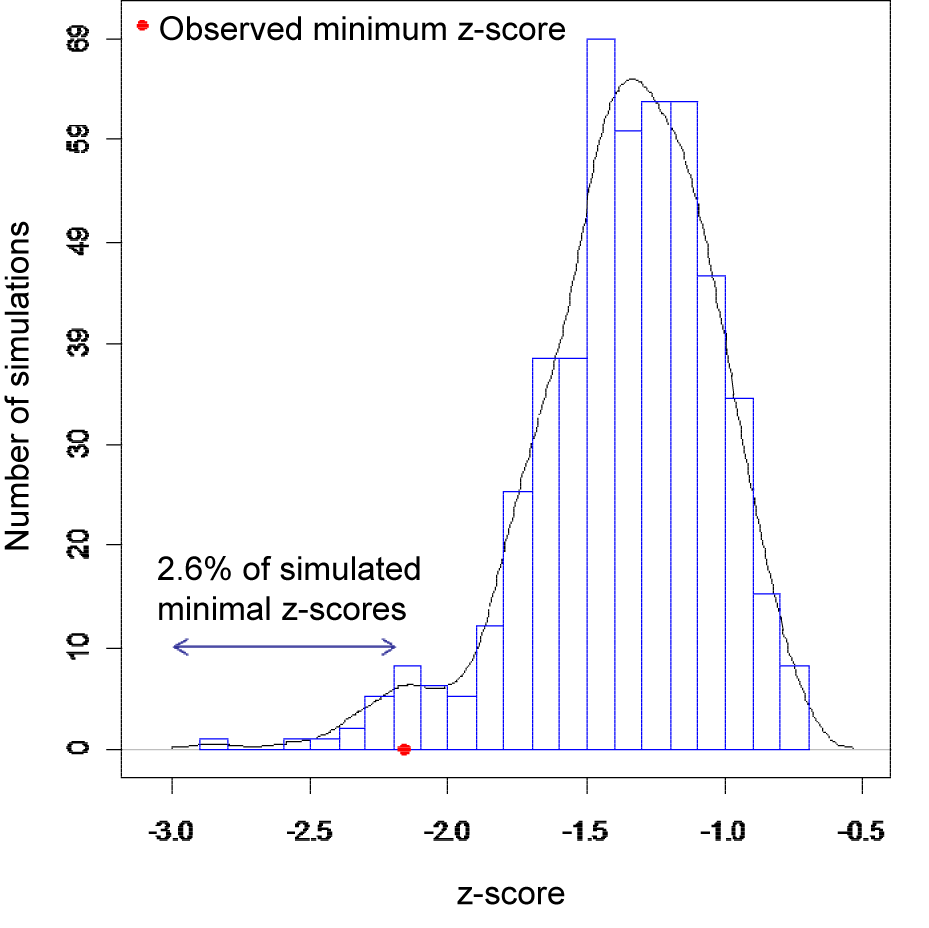

Supplement: Additional file 4 — Simulations - null distribution histogram. The distribution of the lowest z-scores was observed in 500 simulations of selection of the AS-30CQ × AJ cross, using experimental marker positions. The null hypothesis assumed 10 loci for artemisinin resistance and 10 minor fitness alleles. The red point indicates the experimentally observed z-score (-2.364) for chr2 under artemisinin selection (Figure 3A) corresponding to a p-value of 0.0263. See Additional File 2 for details. [file 1471-2164-11-499-S4.TIFF]

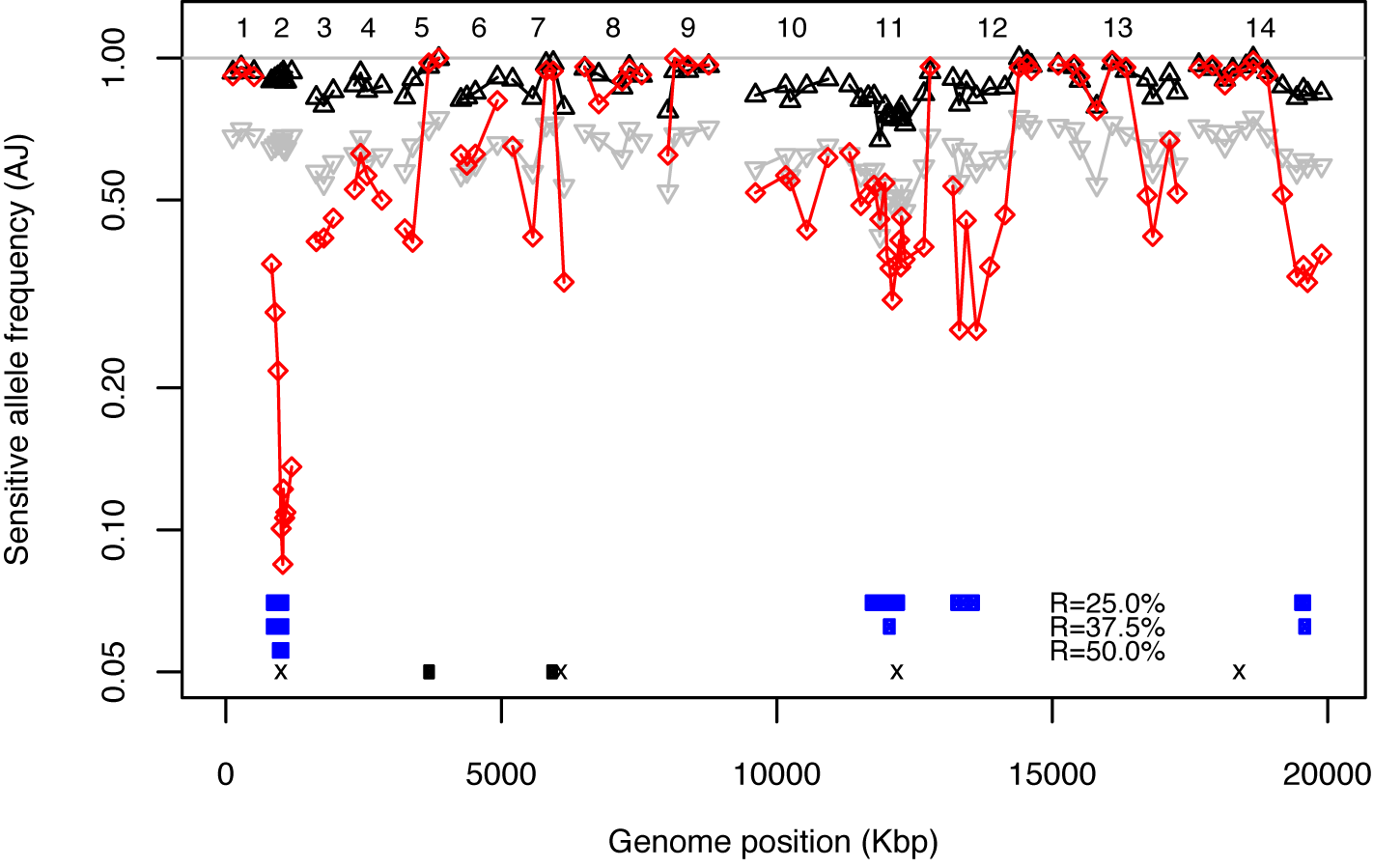

Supplement: Additional file 6 — Mann-Whitney analysis of genome-wide LGS. Scan showing allele frequencies (proportion of AJ allele) from pyrosequencing after untreated infection (control, black triangles) and treated infection (artemisinin 100 mg/kg/day, 3 days, red diamonds) (n.b Figure 3A in main text uses Comparative Index (y-axis, defined in legend Figure 3) rather than allele proportion used here). Treatment is expected to bias AJ allele frequencies downward by removing the sensitive parent, AJ and recombinants free from resistance mutations. To correct for this and other factors (Text S1), control allele frequencies were reduced before testing potential selection valleys. Significant valleys are highlighted by blue circles (P < 0.001 in Mann-Whitney U-tests comparing a sliding window of 3-5 markers on the same chromosome (treated) with all untreated markers reduced by the percentage (R) indicated (AF-reduction); grey triangles show untreated frequencies resulting from a -25% shift). Positions of this strain's new mutations found by genome comparisons are indicated at the bottom (x = SNP; circle = indel). Chromosome numbers are given at the top. Genome position indicates physical mapping (Kbp) of 92 markers in the genome of P. chabaudi (Additional File 1). Data points are each the mean of 3 independent determinations. [file 1471-2164-11-499-S6.TIFF]
