# Supplementary material for: Experimental evolution, genetic analysis and genome re-sequencing reveal the mutation conferring artemisinin resistance in an isogenic lineage of malaria parasites
Source: BMC Genomics. 2010 Sep 16;11:499. doi: 10.1186/1471-2164-11-499 (PMC2996995; doi:10.1186/1471-2164-11-499)
Supplement: Additional file 7 — Solexa whole-genome re-sequencing - extended methods, results, analysis, evaluation and quality control. This file includes an extended description of the analysis of the Solexa Illumina data. [file 1471-2164-11-499-S7.DOC]

**Additional File 7**

**Solexa whole-genome re-sequencing – extended methods, results, analysis, evaluation and quality control**

**Summary**

We present the methods used for analysis of insertion and deletion mutations (indels) and copy number variations (CNVs). We present the confirmed, probable and possible point mutations, small indels, larger indels and CNV mutations identified as common to clones AS-30CQ and AS-15MF. 4 point mutations (chr02, chr07, chr11, chr14) and one 34 bp deletion (chr07) were confirmed by di-deoxy sequencing. We further firmly predict another large deletion (chr05), not presently amenable to further characterisation. We describe our procedures and tests for evaluating point mutations, indels and CNVs below. We also analyse whether we are likely to have missed a mutation, particularly on chr02. Finally we use a simulation to validate these approaches.

**Methods to detect indels and CNVs**

Although both MAQ and SSAHA2 predict ‘small’ indels (here defined as indels ≤ 3 bp) by allowing reads to map when (in the absence of higher quality alignments) either the read or the reference sequence contain gaps of 1, 2 or 3 bp, MAQ reliably provides this feature only when ‘paired-end’ read data are generated. For this reason, only SSAHA2 was used here for small indel prediction. This distinguishes between insertions and deletions and defines the number of nucleotides (1-3 bp) involved (by evaluating ‘gaps’ in AS-WTSI or sequence reads). We discarded small indel calls when less than half of the total reads (mapping a particular candidate) predict an indel. We then classified the reliability of the remaining indels on the basis of the total number of reads and the proportion of reads indicating the predicted indel in SSAHA2.

For the detection of larger indels (> 3 bp), a method based on genome coverage analysis was developed: indels are expected to reduce mapping of reads in these regions. Firstly, read depth was calculated for each base. The read depth of each nucleotide in each mutant clone and in AS-sens was divided by the mean read depth for the whole genome to compute ‘relative coverage’. The relative coverage of each base in the mutant clone was then divided by the corresponding relative coverage in AS-sens, defining a “comparative coverage”. If the comparative coverage < 0.5 (*i.e.* the relative coverage in the mutant clones was less than half the relative coverage in AS-sens) for at least 10 consecutive bases and, additionally, if the absolute read depth in AS-sens was ≥ 20 (to exclude areas where coverage is insufficient (due to systematic bias or random variation) to provide reliable information in AS-sens), a potential indel was called. This method cannot differentiate insertions and deletions (unless low comparative coverage extends over a long stretch of the genome) so that further verification by di-deoxy sequencing is needed for characterisation as well as confirmation. These approaches were successfully evaluated by simulation during this study (see below).

Copy number variations (CNVs) (gene amplification in mutant) were proposed when comparative coverage ≥ 1.5 over at least 200 consecutive nucleotides.

Potential indels of > 3 bp and CNVs (both identified through comparative coverage analysis) were also inspected visually in Artemis and in MAQ. If a large indel or CNV could not be visually verified by MAQ, it was considered less reliable (low quality).

We attempted to identify indels by a comparative coverage analysis (as described for SSAHA2) using MAQ. This proved unreliable (see below): for instance, an excessive number of calls were obtained which were not reproducible after replicate mapping by MAQ.

Potential CNVs were similarly analysed by increased comparative read coverage analysis (mutant relative to sensitive wild-type, see Materials and Methods). No CNVs common to both clones was predicted. However, we were able to confirm the previously reported *mdr1* duplication in AS-15MF [*15*] (data not shown), showing that CNVs may be identified using this methodology.

**Data – description of table contents and format**

Data is given for those calls identified in both AS-30CQ and AS-15MF in Table 2. In Additional Files 8, 9, these data are displayed with each possible mutation colour coded; confirmed (green coded), high confidence (yellow coded) and low confidence (orange coded) for point mutations and indels/CNVs.

These tables include information regarding chromosome number (column B), type of mutation (column C), analysis tool (column D) and nucleotide positions (columns E),

- For point mutations (Additional File 8), additional information includes nucleotide call in reference sequence (F), nucleotide call, read depth and relative coverage for AS-sens (H, I, and K) and AS-30CQ (O, P and Q), SSAHA2 quality index (R) in AS-30CQ.
- For indels (Additional file 9), additional information was nucleotide range (E, F). Read depth refers to a random single base within the mutation. Other information regarding indels and CNVs depends on the nature of the specified indel/CNV.
- For small (≤ 3 bp) insertions or deletions directly predicted by SSAHA2, the type and length are defined (C, G). No read depth is given. Instead, an “indel quality” score, calculated by SSAHA2 is shown (P). This indicates the number of reads calling an indel divided by the total number of reads being mapped to a given base.
- For larger indels and CNVs, read depth, relative and comparative coverage are defined (I, J, L, N and O).
- For point mutations (Additional File 8), the possible biological significance of the mutation may be illuminated by reference to columns U, V, X and Y which define whether mutations are expected to be intra- or inter-genic, synonymous, non-synonymous, intronic, syntenic, non-syntenic, telomeric etc. and which genes are involved.

**Further details**

All the point mutations and two of the indels were identified by MAQ and SSAHA. Small indels were identified by SSAHA2 scores using a filter threshold of > 0.5 of the reads calling an indel.

A number of other analyses were undertaken to investigate the likelihood that SNP mutations were ‘missed’ in our analysis due to low read coverage (‘false negatives’). These are discussed in the following sections

**Point mutations**

Additional File 8 shows that shared point mutations were confirmed on chr02, chr07, chr11 and chr14 (code green). They all have SSAHA quality scores >91 (out of a maximum score of 99).

One striking feature of these data is the small number (four) of point mutations shared by AS-30CQ and AS-15MF. Indeed, even when the AS-30CQ and AS-15MF genomes were considered independently, only 12 and 13 point mutations (respectively) were predicted (data not shown). Of these only 7 and 5 (respectively) are validated high-quality calls. The remaining 5 and 8 (respectively) have low quality scores. SNPs with quality scores such as these are invariably negated by di-deoxysequencing.

We have analysed in detail the likelihood of ‘missing’ a critical mutation, both in the genome and, particularly, within the selection valley on chr02. These we call ‘false negatives’. First, we compared the results obtained from SSAHA2 and MAQ regarding the identification of mutations. Both methods identified the same potential high quality point mutations, which were then confirmed by capillary sequencing (code green). Mutations in SSAHA2 that were not predicted by MAQ and which, in any case, appear only in AS-30CQ or AS-15MF are of low quality and probability (e.g having low read-depth): whenever tested by di-deoxysequencing these have always been negated (data not shown). There were no point mutations that were identified in MAQ but not in SSAHA2. This suggests that we have defined the maximum set of proposed mutations.

False negatives are most likely to arise because of low read depth. Such regions may arise by random sampling, or by systematic variation in read depth, for example, where there are repetitive sequences. In fact, comparison of relative coverage across the genomes of various clones leads us to conclude that much of this variation is systematic (data not shown): the set of regions showing low read depth is reproducible. We analysed the proportions of the genome with low relative coverage, as follows.

The proportion of nucleotides in the mapped AS-WTSI genome with a read depth of ≥ 10 reads in the SSAHA2 data for clone AS-30CQ was 98.31% (Table 4) (range 96.16% (chr01) to 99.39% (chr09)). Chr02 gave 98.92%. Coverage was significantly less extensive in the unassigned contigs (identified as “bin”). This was also observed in AS-SENS and AS-15MF (data not shown) and could indicate the non *Plasmodium* origin of this sequence data, highly repetitive sequence which is difficult to map (e.g. telomeric areas) or areas of low genome sequence quality. Because we considered a minimum read depth of 10 sufficient to call point mutations reliably, the percentage of the genome containing potential false negatives because of low read depth is estimated here to be < ~ 3%. However, it was particularly critical to evaluate the area within the selection valley on chr02. 0.63% of the nucleotides spanning 200 kb either side of *ubp1* show a read depth ≤ 10 (Table 4). This suggests that the percentage of the genome containing potential false negatives in the selection valley is estimated here to be < ~ 1%.

The highest quality of mutation prediction requires adequate read depth but is also enhanced in regions of the genome where sequence reads map uniquely to the reference sequence. We therefore performed an ‘*in silico’* analysis in which all possible 36-mers or 41-mers were mapped to the reference genome when, and only when, mapping defined a unique position in the genome. Table 3 shows that 98.63% and 98.80% of the genome were evaluated (aligned) by uniquely mapping reads when using 36- and 41-mers, respectively. Experimentally, the proportion of the genome for which the read depth of uniquely mapped reads was ≥ 5 (Table 3) was 97.78% and 97.95% for AS-sens and AS-30CQ, respectively. At increased stringency (read depth ≥ 10) these figures were reduced to 95.88% and 96.96%. These data confirm that the proportions of the genome not evaluated by a.) uniquely mappable reads and b.) sufficiently high read-coverage (both major contributory factors to SNP call confidence) are both low <~3%.

**CNV and Indel Detection**

Additional File 9 shows the inventory of indels and CNVs predicted for both AS-30CQ and AS-15MF and evaluated by SSAHA2 and MAQ or by SSAHA2 alone. The possible presence of 5 large indels (on chr01, chr05 and chr07 and two in unassigned contigs (‘bins’)) and 3 small indels (on chr07, chr11 and in an unassigned contig) were indicated in both AS-30CQ and AS-15MF. No CNVs were predicted to be present in both AS-30CQ and AS-15MF.

One small indel (chr11) was negated by di-deoxy sequencing. We confirmed a 34 bp deletion on chr07 and identified with high confidence a deletion(>1 kb) on chr05. We do not expect the two additional small indel calls to be confirmed because of low fold coverage. For the three additional large indel calls, the read-coverages in the mutant clones were not low. These are therefore considered low confidence calls.

There were more potential indels called for either AS-30CQ or AS-15MF alone. These sets of indels (53 and 88, respectively) are large compared to the set of common indel calls. This is strong evidence of a high rate of false positive calls. Still, a comprehensive validation of a large set of small indel calls will be required to establish strict criteria of validity.

Simulation studies (see below) suggest that the false negative rate is low. However, the precise criteria by which false and true positives may be discerned are not clear.

One potential CNV was predicted for AS-30CQ (on chr14) and 3 were predicted for AS-15MF, one of which corresponded to a previously described ~400kb duplication event (15). The two other CNVs are predicted in unassigned contigs (data not shown).

There was no evidence of indels or CNVs in orthologues of three other genes (*atp6, crt*, *tctp*) previously suggested to be involved in artemisinin resistance phenotypes. This is consistent with our previous analysis of these genes in a subsequently selected artemisinin resistant clone, AS-ART [*16*].

**Simulation to evaluate indel analysis**

While CNVs and large indels (> 50 bp) can generally be detected easily by visual analysis of the alignment plots in MAQ and by comparative coverage analysis, intermediate indels (i.e. between 4 bp and 50~ 30 bp) are more difficult to discern. In order to test the reliability of indel detection in SSAHA2, we analysed the mapping (SSAHA2) of sequence read data after the reference genome was modified to include four single nucleotide insertions, four single nucleotide deletions and three 9bp indels (two deletions, one insertion). All 8 single nucleotide indels were reliably detected by the inbuilt SSAHA2 algorithm. Two of the three 9bp indels were identified using the comparative coverage approach developed for this study. This suggests that the strategies used are likely to identify true positives. The missed indel was located in an AT-rich, low complexity region. Thus, while small indels (≤ 3 bp) and large indels (>50bp) can be readily detected, there is the possibility that intermediate indels, especially in low complexity or highly repetitive areas of the genome could be missed. However, it should be also noted that such areas are unlikely to play an active role in the drug resistance phenotype studied here.

A comparative coverage approach was applied to the artificially mutated AS-sens genome using MAQ. Interestingly, one of the single bp indels was missed by the analysis, and all three 9bp indels were detected (cf SSAHA2, above). However, a large number (>1000) of false positives were also called. Replicate mapping (same data against same reference) further revealed that while some false positives were conserved, other false positives appeared or disappeared with each mapping attempt. This rendered a coverage-based indel analysis in MAQ both unwieldy and unreliable for *de novo* indel identification. Visual analysis of coverage in MAQ was however still used to support CNVs and indels detection by SSAHA2.
