# Supplementary material for: Experimental evolution, genetic analysis and genome re-sequencing reveal the mutation conferring artemisinin resistance in an isogenic lineage of malaria parasites
Source: BMC Genomics. 2010 Sep 16;11:499. doi: 10.1186/1471-2164-11-499 (PMC2996995; doi:10.1186/1471-2164-11-499)
Supplement: Additional file 11 — AS-15CQ genotype and the origin of alternative ubp1 V2697F mutation in AS-ATN. This file discusses a number of issues regarding AS-15CQ, its non-clonality and the origins of two mutations in ubp1. [file 1471-2164-11-499-S11.doc]

**Additional File 11**

**AS-15CQ genotype and the origin of alternative *ubp1* V2697F mutation in AS-ATN**

***Ubp1* mutations**

Following the discovery of *ubp1* V2728F (formerly V770F) in AS-ART and AS-30CQ, genotyping of AS-ATN revealed a different V2697F mutation (formerly V739F) along with the wildtype V2728 allele [27]. AS-15CQ, when originally tested, gave a wild type haplotype (V2698, V2727). We therefore previously assumed that 2697F originated and was selected by artesunate selection of AS-15CQ and that 2728F arose by chloroquine selection of the same parasite [27].

Note that both V2697F and V2728F mutations were predicted to reduce the activity of the de-ubiquitinating enzyme encoded by this gene; the former by distorting the active site, the latter by reducing substrate (ubiquitin moiety) binding (distortion of a hydrophobic pocket forming a critical contact with ubiquitin) [27].

**Origin of *ubp1* mutations and artemisinin resistance**

This raised a number of problems regarding role of *ubp1* mutations in artemisinin and artesunate resistance (fully discussed in [27] and the current text). Furthermore, subsequent determination of *ubp1* haplotype in AS-15CQ gave ambiguous and conflicting results depending upon the sample of AS-15CQ used as template. All alleles, 2697V,F and 2728V,F could be variously detected at different proportions in different cryopreserved stabilates and after growth of parasites under different drug conditions (data not shown).

In fact, AS-15CQ is uncloned (although bottlenecked during mosquito transmission) after passage of AS-3CQ with chloroquine selection [14]. It must therefore be considered a mixed population of parasites. One consequence is that (fixed) mutations appearing in those clones derived from AS-15CQ by selection under chloroquine (AS-30CQ) [14], mefloquine (AS-15MF) [15], or artesunate (AS-ATN) [16] may (or may not) be detectable in AS-15CQ in various and varying proportions.

We resolve these data as follows:-

Both *ubp1* mutant alleles (2697F and 2728F) are present in AS-15CQ (in different parasites). The haplotypes present are therefore wild-type (2697V, 2728V) and mutants (2697F, 2728V and 2697V, 2728F). Both mutant alleles were selected (but not to fixation) by chloroquine treatment (between AS-3CQ and AS-15CQ). Subsequent passage, under mefloquine or chloroquine treatment, specifically selected (or randomly cloned) the 2697V, 2728F mutated haplotype in AS-15MF and AS-30CQ, respectively. In contrast, artesunate treatment specifically selected (or randomly cloned) the 2697F, 2728V mutated haplotype.

This suggests that *ubp1* 2728F was selected by chloroquine (AS-3CQ to AS-30CQ) and by chloroquine and mefloquine (AS-3CQ to AS15-MF). The *ubp1* 2697F mutation was selected by chloroquine and artesunate (AS-3CQ to AS-ATN).

It also suggests that other mutations identified in AS-30CQ, AS-15MF and AS-ATN (relative to AS-3CQ), such as a AS-15MF-specific point mutation, referred to in the main text, may also have been selected prior to AS-15CQ during chloroquine treatment.
